# Development of the Workplace Interpersonal Problems Scale for Care Workers (WIPS) and examination of its reliability and validity

**DOI:** 10.1016/j.heliyon.2023.e20156

**Published:** 2023-09-16

**Authors:** Shinya Takeda, Toshiki Fukuzaki

**Affiliations:** Department of Clinical Psychology, Tottori University Graduate School of Medical Sciences, Japan

**Keywords:** Workplace Interpersonal Problems Scale for Care Workers, Content validity, Construct validity, Structural validity, Test–retest reliability

## Abstract

**Introduction:**

The turnover rate of care workers has remained high by global standards, with previous studies showing an association between workplace interpersonal relations and care worker turnover and turnover intentions. This study details the development of the Workplace Interpersonal Problems Scale for Care Workers (WIPS) and examines its reliability and validity according to the COSMIN guidelines.

**Methods:**

A total of 476 care workers employed by elder care facilities participated in the study. This study examined the reliability and validity of the WIPS after its development. Reliability was evaluated using Cronbach's α, test reliability with the standard error of measurement, and test–retest reliability with the intraclass correlation coefficient. Content validity, construct validity, and structural validity were examined to evaluate validity.

**Results:**

Both total and subscale scores of the WIPS had a Cronbach's α coefficient >0.75 and high test–retest reliability (intraclass correlation coefficient, 0.75). Content validity analysis showed the item-content validity index of ≥0.90 for all WIPS items, confirming 100% of the hypotheses for testing construct validity. Confirmatory factor analysis showed an acceptable fit for the hypothesized six-factor construct (CFI = 0.92, TLI = 0.91, RMSEA = 0.07, SRMR = 0.05).

**Conclusions:**

The WIPS was found to be a valid and reliable instrument. With the growth of the elderly population worldwide, we believe that the WIPS will be a useful quantitative measure to assess workplace interpersonal problems affecting care workers in various aspects.

## Highlights

• The WIPS is an instrument developed to assess workplace interpersonal problems among care workers working in elder care facilities.
• The reliability and validity of the WIPS were confirmed based on the Consensus-based Standards for the Selection of Health Measurement Instruments guidelines.
• With the growth of the elderly population worldwide, we believe that the WIPS will be a useful quantitative measure to assess workplace interpersonal problems affecting care workers in various aspects.

## 1 Introduction

The elderly population is expected to grow rapidly worldwide [1], with such an increase having been associated with an increase in the number of people suffering from various chronic diseases, including dementia, who could be requiring nursing care. Therefore, care workers who provide care for the elderly are expected to play an increasingly important social role in the future. However, the shortage of care workers has been a persistent global problem [2], with studies showing high turnover rates among care workers [3]. High turnover and understaffing among care workers reduce the quality of caregiving and organizational efficiency [4,5]. Therefore, preventing care worker turnover is a critical issue worldwide given the expected rapid increase in the elderly population. Considering the close relationship between turnover and turnover intentions [6], addressing factors that increase turnover intentions may be useful to prevent care worker turnover.

In Japan, being a care worker is a profession on its own. Although getting certified as a care worker is required for those working in elder care facilities, it is still possible for individuals to work as care workers without certification [7]. Care workers in elder care facilities are responsible for providing physical assistance like eating, bathing, and toileting, daily living support such as cleaning and laundry, support for social activities such as leisure time activities, and consultation for health issues. They play an important role in reducing the burden on the healthcare system and promoting a team approach to healthcare by providing essential care to the elderly in need.

A factor consistently shown to be associated with care worker turnover and turnover intentions in Japan has been workplace interpersonal problems [7]. Previous studies have shown that workplace interpersonal relations are associated with turnover and turnover intentions among care workers worldwide. For example, care workers with workplace interpersonal problems were found to be around twice as likely to have higher turnover intentions than were care workers without such problems [8]. Supervisor satisfaction has been reported to be negatively associated with turnover intentions [9]. Lack of support and communication from supervisors and coworkers has also been found to be associated with high turnover intentions [10]. Care workers responsible for caring for the elderly have shown that increased perception of teamwork in the workplace lowers the turnover intention and improves quality of care [11].

On the other hand, workplace interpersonal relations also affect communication among staff [12], and good communication among staff increases quality of care and job satisfaction [13,14]. Support and recognition from colleagues and praise and approval from supervisors have also been shown to lower care worker turnover and improve staff mental health [3,15,16]. Good leadership has been shown to increase care workers' affective organizational commitment [17], which can lower turnover intentions [18]. This suggests that appropriate guidance of subordinates and newcomers significantly impacts the retention of care workers in the workplace. Studies on the relationship between interpersonal relationships in work settings and burnout among home care workers have shown that conflict with supervisors was significantly associated with emotional exhaustion, whereas conflict with coworkers was significantly associated with depersonalization [19]. Meanwhile, poor workplace interpersonal relations can lead to problems such as bullying, which not only facilitates turnover intentions and turnover [20] but also decreases the quality of care [21]. As such, workplace interpersonal problems among care workers are an important factor associated with not only turnover intentions but also care workers' mental health and quality of care.

Available evidence suggests that assessing workplace interpersonal problems among care workers should be a priority in elder care facilities. To date, only one scale has been developed to assess workplace relationships among nursing staff working in university hospitals, which assesses interpersonal relations based on 29 items with 6 factors [22]. However, the scale developed by Dias et al. focuses on the physical and mental fatigue brought on by interpersonal relations and the behaviors and emotions that affect interpersonal relations and not on the content of workplace interpersonal problems experienced by care workers. In addition, no quantitative measure of workplace interpersonal problems is available for care workers working in elder care facilities. This approach would help prevent care workers from leaving their jobs and maintain and improve their mental health, as well as contribute to improving the workplace environment and quality of care for elderly people needing nursing care.

In this study, we detail the development of the Workplace Interpersonal Problems Scale for Care Workers (WIPS), which quantitatively assesses workplace interpersonal problems experienced by care workers working in elder care facilities, and examine its reliability and validity. The WIPS is based on the Consensus-based Standards for the Selection of Health Measurement Instruments (COSMIN) [[23], [24], [25]].

## 2 Material and methods

### 2.1 Participants

The previous study found that workplace interpersonal problems are consistently linked to care worker turnover and turnover intentions in Japan [7]. Therefore, this study focused on independent professionals who took care of elderly individuals and was conducted among registered members of a large Internet survey company in Japan. Care workers were invited to take the online survey based on their registered member information. To ensure accuracy, a screening item was used to confirm whether the member was currently employed as a care worker at an elder care facility. The target number of respondents for the first survey (T1) was set at the COSMIN standard of 7 times the number of items and at least 100 respondents [23]. The COSMIN [23] determines whether a patient-reported outcome measure (PROM) development study was conducted with a sample representative of its target population. A large survey in Japan conducted in 2021 [26] showed that the overall age of care workers ranged from 20s to 60s (88.1%). Therefore, we attempted to draw a sample of 50 men and 50 women for each 10-year age bracket from their 20s–60s, for a total of 500 respondents. We were able to identify 50 men and 50 women across all age brackets, except for men in their 20s, of whom only 26 were available. Those who did not satisfy the response time standard set by the Internet survey company (i.e., those who left the response screen empty for an extended period and did not respond) and those who did not correctly answer trap questions that required responses to specific options (i.e., “please answer the bottom option for this question”) were excluded from the data. Ultimately, we collected responses from 476 individuals at T1 (226 males and 250 females; mean age 45.5 ± 13.0 years). For the second survey (T2) to confirm retest reliability, half of the subjects were selected from those in T1. The age and gender were set to be in the same proportions as in T1; since T1 did not meet the target collection for males in their 20s, as many as possible of the 26 subjects collected in T1 were collected for T2. As a result, 16 males in their 20s were collected for T2, for a total of 241 (116 males and 125 females, mean age 45.2 ± 13.0 years). All data were collected from September to October 2022.

### 2.2 WIPS items generation

The WIPS items were developed by two researchers specializing in the mental health of care workers using the following steps. In our study [8], six factors were identified as workplace interpersonal problems faced by care workers employed by elder care facilities: “inadequate communication,” “bullying,” “sense of unfair workload,” “different attitudes to care work,” “difficulty in guidance for subordinates/new staff,” and “labeling.” According to a large survey in Japan, the top workplace interpersonal problems faced by care workers working in elder care facilities were “difficulty in supervising subordinates,” “supervisors and coworkers who do not agree with me,” “insufficient exchange of opinions on care methods,” “poor management skills of management, managers, etc., and unclear or insufficient work instructions,” and “difficulty communicating with supervisors and coworkers” [27]. These factors were associated with the six factors we identified because they are consistent with the care workers response that comprise each factor found in our study [8]. In addition, the workplace interpersonal problems of care workers described in the Introduction section of the current paper were associated with the six factors we identified. Based on the mentioned data, we determined that the six factors identified reached a certain level of saturation in terms of workplace interpersonal problems faced by care workers working in elder care facilities, based on which we developed the items using descriptive data obtained from our study [8] on workplace interpersonal problems as identified by care workers. The descriptions of care workers related to the six factors were comprehensively addressed, and similar descriptions were combined into one item. Consequently, the WIPS was completed with 23 items: 4 items for “inadequate communication,” 4 items for “bullying,” 3 items for “sense of unfair workload,” 4 items for “different attitudes to care work,” 4 items for “difficulty in guidance for subordinates/new staff,” and 4 items for “labeling” (Table 1). Hence, we believe that a certain level of comprehensiveness, a measure of whether a critical aspect of the construct is missing, was maintained as required by the COSMIN guidelines [23]. The participants read each question while thinking about interpersonal relations at their workplace and chose the number that most applies to their situation in the past 2 weeks. The reason for the 2 weeks setting was our belief that this period would capture the participants' usual workplace interpersonal problems since there would be no significant changes in the workers' work habits during this period [28]. All items are based on a 4-point Likert-type scale (0 = not at all applicable, 1 = not applicable enough, 2 = slightly applicable, 3 = very applicable). In conducting statistical analysis, this scale's overall and subscale scores were used as the total score for each question item.

**Table 1:** WIPS items and results of WIPS confirmatory factor analysis and I-CVI

		Standardized factor loadings						I-CVI
	WIPS items	Insufficient communication	Bullying	Sense of unfair workload	Different attitudes to care work	Difficulty in guidance for subordinates/new staff	Labeling	
Insufficient communication								
	I have felt like my opinions are ignored.	0.77						1.00
	I have had a hard time expressing what I wanted to say to my coworkers.	0.77						1.00
	There are times when I did not feel comfortable asking questions or consulting with my supervisors or coworkers.	0.76						1.00
	I have not communicated well enough with my coworkers.	0.63						1.00
Bullying								
	There have been times when my coworkers talked to me in a passive-aggressive manner.		0.83					1.00
	I have been unreasonably blamed by my coworkers in a strong tone of voice.		0.74					0.90
	There have been times when I have been ignored by my coworkers.		0.64					0.90
	I have overheard my coworkers talking bad about other coworkers, even though I didn't want to hear it.		0.57					0.90
Sense of unfair workload								
	I have been frustrated when my workload differed from that of my coworkers due to my supervisor's inadequate management skills and directions.			0.83				1.00
	I have felt that I was treated unfairly because my workload differs from that of my coworkers.			0.77				1.00
	I have felt that I was the only person working hard while some coworkers were just relaxing.			0.73				1.00
Different attitudes to care work								
	I have had difficulties with my coworkers due to disagreements on the future directions at work.				0.77			1.00
	I have had difficulties with my coworkers due to differences in perspectives based on rank or post.				0.74			0.90
	I have had difficulties with my coworkers due to differences in perspectives because of an age gap.				0.68			0.90
	I have had difficulties with my coworkers due to differences in our views regarding caregiving and care.				0.60			1.00
Difficulty in guidance for subordinates/new staff								
	I have had difficulties due to differences in values between myself and my subordinates or new staff.					0.81		0.90
	I have had difficulties mentoring subordinates or new staff due to disagreements in opinions.					0.72		1.00
	I have had difficulties mentoring subordinates or new staff.					0.70		1.00
	There have been times when I did not know how to talk to my subordinates or new staff.					0.67		0.90
Labeling								
	When a disagreement occurred with my coworkers, I have thought, “This happened because I didn't get along with this person.”						0.86	1.00
	When a disagreement occurred with my coworkers, I have thought, "This happened because I am not fond of this person."						0.84	1.00
	When a disagreement occurred with my coworkers, I have thought, “This happened because of a person's bad personality."						0.73	1.00
	I have negatively labeled my coworkers.						0.70	1.00
Covariance								
	Insufficient communication	–						
	Bullying	0.86	–					
	Sense of unfair workload	0.88	0.74	–				
	Different attitudes to care work	0.96	0.84	0.94	–			
	Difficulty in guidance for subordinates/new staff	0.86	0.70	0.81	0.88	–		
	Labeling	0.85	0.73	0.80	0.79	0.81	–	

Notes: WIPS: Workplace Interpersonal Problems Scale for Care Workers; I-CVI: Item-Content Validity Index.

### 2.3 Measures

The following scales were administered to examine construct validity. The “Interpersonal stress at workplace,” a subscale of the Brief Job Stress Questionnaire [29], was used to measure workplace interpersonal problems. This scale consists of three items, with higher scores indicating greater workplace interpersonal stress. Questions included items such as “There are differences of opinion within my department.” Participants were asked to answer these questions using the four-point scale (1 = very much so, 2 = moderately so, 3 = somewhat, 4 = not at all). To measure support in the workplace, we used the “Supervisor support” and “Co-worker support” subscales of the Brief Job Stress Questionnaire [29]. These scales consist of three items, with higher scores indicating greater support from supervisors and coworkers. Participants were asked to answer questions such as, "How freely can you talk with the following people?" and to imagine their supervisors and coworkers and answer these questions. Participants were asked to respond on a four-point scale (1 = extremely, 2 = very much, 3 = somewhat, 4 = not at all). To measure job satisfaction, we used the Japanese version of the NIOSH Generic Job Stress Questionnaire [30] consisting of four items, with higher scores indicating greater job satisfaction. Questions include "If you had to decide whether or not to do your current job again, what would you do?" and so on. Respondents were asked to answer this question using a three-point scale (1 = decide to stay in the same job without hesitation, 2 = consider another job, 3 = never stay in this job). Three of the four questions require a three-point scale, but one requires a four-point scale. This question asked, "Overall, how satisfied would you say you are with your job?" The responses were as follows (1 = very satisfied, 2 = somewhat satisfied, 3 = not very satisfied, 4 = not satisfied at all). To measure psychological distress, we used the Japanese version of the K6 scale [31] consisting of six items, with higher scores indicating higher psychological distress. Questions included “During the last 30 days, how often did you feel nervous?” and others, with a five-point scale of (0 = none of the time, 1 = a little of the time, 2 = some of the time, 3 = most of the time, 4 = all of the time). Turnover intention was measured using the turnover intention scale [32,33], which includes one reversal item out of four items. However, Sakakibara et al. (2020) [34] showed that the inclusion of this reversal item significantly reduces the Cronbach's α coefficients. Therefore, the current study used the three items excluding the reversal item, similar to that in Sakakibara et al. (2020) [34], with higher scores indicating higher turnover intention. The questions included items such as "I consider my decision to work for this employer as an obvious mistake,” and responses were given on a five-point scale (1 = I agree completely, 2 = somewhat agree, 3 = neither agree nor disagree, 4 = somewhat disagree, 5 = I disagree completely). For all of these scales above, total scores were used for statistical analysis.

The aforementioned measures and information on age, sex, marital status, education, career in the current job (year), years of experience as a care worker, employment status, care giving office, managerial position, and qualification were collected during the survey at T1.

### 2.4 Test–retest reliability

To confirm test–retest reliability, the WIPS was performed again two weeks (T2) after the first run (T1). According to the COSMIN guidelines, adequacy of time intervals to assess stability between measurement points is required [23]. An interval of 2–4 weeks is the most recommended for test–retest reliability [35]. Evidence also suggests that 2 weeks is sufficient to ensure no change in respondent's work performance [28]. Therefore, a two-week interval was used to confirm test–retest reliability. Only respondents who reported no change in position or department between T1 and T2 and those who reported that they had not left their work were included in determining test–retest reliability.

### 2.5 Content validity

Content validity is defined as the degree to which items in an assessment scale are relevant to and representative of the targeted constitutive concept for a particular evaluation purpose [36]. In addition to comprehensiveness mentioned previously, relevance, which indicates the extent to which all questionnaire items are related to the construct being measured, has been identified as a component of content validity based on the COSMIN guidelines [25]. The most commonly used method for quantitatively calculating content validity with respect to relevance is the Content Validity Index (CVI) [36]. Accordingly, the Item-CVI (I-CVI) is a four-point scale in which multiple experts rate the degree to which each question item is related to the construct being measured using the following options: “not related,” “somewhat related,” “fairly relevant,” and “very relevant.” The I-CVI value is calculated by determining the number of experts who rated each item as “very relevant” and dividing it by the total number of experts [37]. However, given that this four-case method of evaluation can be arbitrary [38], the current study calculated the I-CVI as follows. First, six factors included in the WIPS were explained in writing to the expert conducting the evaluation. Thereafter, they were instructed to determine which of the six factors each of the 23 items corresponded to using the statement "Each of the following questions asks about the workplace interpersonal problems of care workers. Which of the six factors do you think each question applies to, please choose only one." Similar to the study by Yusoff (2019) [36], the current study selected 10 experts, consisting of 6 university faculty members with research experience in elder care or mental health and 4 psychiatrists with over 10 years of clinical experience in elder care or mental health, to conduct the evaluation.

### 2.6 Construct validity

Construct validity was assessed using COSMIN hypothesis testing guidelines. The COSMIN guidelines define construct validity as the degree to which a developed PROM is consistent with a hypothesis regarding its relationship to other PROMs [23]. Previous studies have found that workplace interpersonal problems among care workers were associated with psychological distress [8], turnover intentions [8,16], job satisfaction [9,39], supervisor support [10,15], and coworker support [14,40]. Therefore, the hypotheses presented in Table 2 are based on the mentioned findings.

**Table 2:** Hypotheses on construct validity.

	Hypotheses	Rationale
1	There will be a high positive correlation between WIPS and Interpersonal relationship.	WIPS and interpersonal relationship, one of the BJSQ subscales [29] measure similar constructs but asked differently.
2	There will be a moderate positive correlation between WIPS and K6.	Psychological distress has been identified in literature as a risk factor for workplace interpersonal relations problems among care workers [8].
3	There will be a moderate positive correlation between WIPS and intention to leave the organization scale.	Turnover intention has been identified in literature as a risk factor for workplace interpersonal relations problems among care workers [8, 16].
4	There will be a moderate negative correlation between WIPS and job satisfaction.	Leadership and communication have been identified in the literature as being associated with job satisfaction of nursing staff. Leadership and communication are associated with workplace interpersonal relations problems among nursing staff [9, 39].
5	There will be a moderate negative correlation between WIPS and supervisor support.	Support from supervisor are associated with workplace interpersonal relations and has been identified in the literature as a risk factor for turnover intention among care workers [10, 15].
6	There will be a moderate negative correlation between WIPS and co-worker support.	Support from co-worker are associated with workplace interpersonal relations and has been identified in the literature as a risk factor for turnover intention among care workers [14, 40].

Notes:WIPS: Workplace Interpersonal Problems Scale for Care Workers; BJSQ: Brief Job Stress Questionnaire; K6: Kessler 6-item Psychological Scale.

Based on the COSMIN guidelines [24], an |r| of 0.30–0.50 indicates a moderate correlation, whereas an |r| of ≥0.50 indicates a strong correlation. The COSMIN guidelines states that correlations with instruments measuring similar constructs should be strong (i.e., ≥0.50), whereas correlations with instruments measuring related but dissimilar constructs should be moderate (i.e., 0.30–0.50) [24]. Therefore, we considered hypothesis 1 to indicate a strong correlation and hypotheses 2 through 6 to indicate a moderate correlation. Accordingly, construct validity can be confirmed if ≥ 75% of the hypotheses are accepted [24].

### 2.7 Statistical analysis

The Kolmogorov–Smirnov test was performed to determine whether the total WIPS scores were normally distributed. First, the mean, standard deviation (SD) of the WIPS and subscales scores were calculated. Thereafter, Cronbach's α coefficients were calculated to verify internal consistency. The intraclass correlation coefficient (ICC) and standard error of measurement (SEM) were also calculated to confirm test–retest reliability. Second, confirmatory factor analysis was conducted to examine structural validity. The goodness-of-fit of the six-factor structures extracted by Takeda and Fukuzaki (2023) [8] was examined by calculating CFI, TLI, RMSEA, and SRMR. Finally, to examine construct validity, we calculated Pearson's correlation coefficient for the associations between the WIPS and its subscales and interpersonal stress at work, supervisor support, coworker support, job satisfaction, psychological distress, and turnover intentions.

## 3 Results

### 3.1 Attributes

Table 3 shows the demographic characteristics of the study population at T1 and T2. Although we planned to include an equal number of male/female participants across each age bracket, we failed to include enough males in their 20s. Hence, approximately 48% and 52% of the participants were male and female at both baseline and follow-up, respectively, with those in their 20s accounting for approximately 16% or 17% of the participants and the other age groups accounting for 21% each. No differences in the distribution of demographic characteristics were observed between the baseline and follow-up surveys. The highest percentage of respondents in terms of education and employment were high school graduates and formal employees. Around 70% of the caregiving office was residential services at both T1 and T2. Employee status was full-time for about 90% of both T1 and T2. In both T1 and T2, about 70% of the participants held the certified care worker credential.

**Table 3:** Demographic characteristics of participants.

		The first survey (T1)		The second survey (T2)	
		*n*	％	*n*	%
Gender					
	Men	226	47.5	116	48.1
	Women	250	52.5	125	51.9
Age					
	20–29 years old	76	16.0	41	17.0
	30–39 years old	100	21.0	50	20.7
	40–49 years old	100	21.0	50	20.7
	50–59 years old	100	21.0	50	20.7
	60–69 years old	100	21.0	50	20.7
	Mean ± S.D.	45.5 ± 13.0		45.2 ± 13.0	
Marital status					
	Unmarried	173	36.3	94	39.0
	Married	209	43.9	100	41.5
	Divorce	85	17.9	43	17.8
	Bereavement	9	1.9	4	1.7
Education					
	University/graduate school graduate	163	34.2	78	32.4
	Vocational school/college graduate	131	27.5	65	26.9
	High school graduate	177	37.2	95	39.4
	Junior high school graduate	5	1.1	3	1.2
Career in the current job (yrs)					
	Mean ± S.D.	6.4 ± 5.9		6.2 ± 5.6	
Years of experience in care worker (yrs)					
	Mean ± S.D.	10.0 ± 6.6		10.2 ± 6.8	
Employee status					
	Full-time	429	90.1	221	91.7
	Part-time	47	9.9	20	8.3
Care giving office					
	Home services	147	30.9	66	27.4
	Residential services	329	69.1	175	72.6
Managerial position					
	No	368	77.3	193	80.1
	Yes	108	22.7	48	20.0
Qualification					
	No	143	30.0	75	31.1
	Yes	333	70.0	166	68.9

### 3.2 Reliability

Table 4 shows the mean, SD, Cronbach's α coefficient, ICC, and SEM for WIPS total and subscale scores. The Cronbach's α values for the overall WIPS score and each subscale score were 0.95 and 0.78–0.86, respectively. On the other hand, the ICC values for the overall WIPS score and each subscale score were 0.75 and 0.63–0.72, respectively. The SEM values for the overall WIPS score and each subscale score were 7.67 and 1.42–1.78, respectively. These results indicate that WIPS is a measure with good internal consistency and retest reliability.

**Table 4:** Means, SD, Cronbach's α, ICC, and SEM on WIPS.

		Mean	SD	Cronbach's α	ICC (95%CI)	SEM
WIPS (total）		51.7	14.6	0.95	0.75 (0.69–0.80)	7.67
WIPS（subscales）						
	Insufficient communication	8.9	2.9	0.83	0.70 (0.63–0.76)	1.69
	Bullying	8.3	2.9	0.78	0.70 (0.63–0.76)	1.62
	Sense of unfair workload	7.3	2.5	0.82	0.71 (0.64–0.77)	1.42
	Different attitudes to care work	9.4	2.7	0.79	0.70 (0.63–0.76)	1.66
	Difficulty in guidance for subordinates/new staff	8.9	2.9	0.82	0.63 (0.55–0.70)	1.78
	Labeling	8.9	3.1	0.86	0.72 (0.65–0.77)	1.67

Notes: WIPS: Workplace Interpersonal Problems Scale for Care Workers; SD: Standard deviations; ICC: Intraclass correlation coefficients; SEM: Standard error of measurement.

### 3.3 Validity

The I-CVI, an index of content validity, was calculated by 10 experts. Accordingly, the results showed that all items had an I-CVI of ≥0.90 (Table 1). In other words, more than 9 of the 10 experts were able to correctly match each questionnaire item to its corresponding factor.

Pearson's correlation coefficients between the WIPS and each instrument used to measure construct validity are shown in Table 5. A strong positive correlation was found for between the WIPS and interpersonal stress at workplace, which measures constructs similar to the WIPS. All correlations between the WIPS and instruments measuring related but dissimilar constructs showed moderate correlations. Among these instruments, supervisor support and coworker support, as well as job satisfaction, showed a moderate negative correlation with the WIPS. Psychological distress and turnover intention showed moderate positive correlations. Hypothesis testing showed that all six hypotheses were accepted (100%). On the other hand, the WIPS subscale scores showed a slightly different trend from the WIPS total score in the following factors showed. Notably, insufficient communication showed a strong negative correlation with supervisor support and coworker support, whereas bullying showed a weak negative correlation with job satisfaction. Difficulty in guidance for subordinates/new staff showed a weak negative correlation with job satisfaction and a weak positive correlation with turnover intention.

**Table 5:** Pearson's correlation coefficients for WIPS and its subscales and other relevant variables.

		WIPS（total）	WIPS（subscales）					
			Insufficient communication	Bullying	Sense of unfair workload	Different attitudes to care work	Difficulty in guidance for subordinates/new staff	Labeling
WIPS（subscales）								
	Insufficient communication	0.90						
	Bullying	0.83	0.71					
	Sense of unfair workload	0.85	0.72	0.63				
	Different attitudes to care work	0.89	0.78	0.70	0.75			
	Difficulty in guidance for subordinates/new staff	0.84	0.69	0.58	0.65	0.70		
	Labeling	0.86	0.72	0.63	0.68	0.67	0.69	
	Interpersonal stress at workplace	0.56	0.54	0.48	0.48	0.54	0.38	0.45
	Supervisor support	−0.48	−0.52	−0.37	−0.43	−0.46	−0.34	−0.37
	Co-worker support	−0.44	−0.52	−0.32	−0.32	−0.42	−0.30	−0.41
	Job satisfaction	−0.36	−0.36	−0.23	−0.37	−0.32	−0.25	−0.33
	Psychological distress	0.43	0.42	0.35	0.35	0.37	0.35	0.38
	Turnover intention	0.44	0.46	0.36	0.43	0.42	0.28	0.34

Note: All correlation coefficients are significant (p < .001.).

WIPS: Workplace Interpersonal Problems Scale for Care Workers.

Confirmatory factor analysis showed that the standardized factor loadings were greater than 0.50 and that all factors were significant (Table 1). Furthermore, the covariance among the factors was greater than 0.70, all of which were significant. Goodness-of-fit indices showed that the hypothesized six-factor structure had a relatively acceptable fit (CFI = 0.92, TLI = 0.91, RMSEA = 0.07, SRMR = 0.05).

## 4 Discussion

The current study aimed to develop and examine the reliability and validity of the WIPS based on the COSMIN guidelines [[23], [24], [25]]. First, according to the COSMIN guidelines [23], a sample size of at least 7 times number of items and 100 or more participants is required to adequately examine the reliability and validity of a newly created scale. In the present study, both T1 and T2 exceeded the mentioned criteria, suggesting that the sample size was adequate. However, only male participants in their 20s did not reach the target of 50 at T1. According to a large survey in Japan, care workers in their 20s account for the smallest proportion (6.5%) of all care workers, with 18.8% and 79.4% of care workers being male and female, respectively [26]. Based on the attributes of Japanese care workers, the small sample size of men in their 20s is also a reasonable result.

Regarding the internal consistency of the WIPS, both the WIPS total and subscale scores showed Cronbach's α coefficients above 0.75. For test–retest reliability, the WIPS total score was fairly stable at 2 weeks (ICC, 0.75). According to the COSMIN guidelines, a Cronbach's α coefficient and ICC of at least 0.7 for each unidimensional scale or subscale indicates sufficient internal consistency and test–retest reliability [24]. The aforementioned findings therefore confirmed that the WIPS demonstrated a sufficient reliability.

Content validity analysis revealed that all WIPS items had showed an I-CVI of ≥0.90. Evidence suggest that an I-CVI of >0.79, which can range from 0 to 1, indicates that the questionnaire is relevant to the construct it is trying to measure [37]. Thus, the aforementioned findings confirm that each item in the WIPS was associated with workplace interpersonal problems among care workers working in elder care facilities, indicating the high content validity of the WIPS.

To examine construct validity, six hypotheses were developed for this study. According to the COSMIN guidelines, the construct validity of a scale is sufficient when 75% of the hypotheses are accepted [24]. In addition, the same criteria recommend that correlations with instruments measuring similar constructs should have a coefficient of at least 0.50 and that correlations with instruments measuring related but dissimilar constructs should have coefficients ranging from 0.30 to 0.50. In the present study, all hypotheses formulated to examine construct validity had been accepted (100%). A strong correlation (i.e., 0.56) was observed between the WIPS and a similar construct, interpersonal stress at workplace, whereas the correlations between the WIPS and the other instruments were all moderate (between + or - 0.30 and + or - 0.50.). Thus, the WIPS was confirmed to be a scale with sufficient construct validity. Supervisor and coworker support has been found to be a predictor of burnout among care workers employed by elder care facilities [41]. In addition, higher levels of psychological distress and burnout and lower job satisfaction have been associated with higher turnover intentions among healthcare workers [42]. In the present study, the WIPS showed moderate negative correlations with supervisor and coworker support and job satisfaction and moderate positive correlations with psychological distress and turnover intention. Therefore, these findings suggest that generous support from supervisors and coworkers is necessary to prevent psychological distress and burnout and improve job satisfaction among care workers, which are closely related to turnover intentions. For each subscale, a strong negative correlation was found between insufficient communication and supervisor and coworker support. On the other hand, bullying had a weaker correlation with job satisfaction than with the other subscales, whereas difficulty in guidance for subordinates/new staff had a weaker correlation with job satisfaction and turnover intention than with the other subscales. These results suggest the need to work on different areas of workplace interpersonal problems among care workers in order to improve psychological factors, such as job satisfaction and turnover intentions, and environmental factors, such as supervisor and coworker support.

Structural validity refers to the degree to which the scores of a PROM adequately reflect the dimensionality of the construct to be measured [43]. The COSMIN guidelines strongly recommend that structural validity be verified by conducting confirmatory factor analysis [23]. In the current study, confirmatory factor analysis was conducted assuming that WIPS had the six-factor structure identified by Takeda and Fukuzaki (2023) [8] as workplace interpersonal problems for care workers. The results showed that the goodness-of-fit indices had relatively acceptable values for the six-factor structure (CFI = 0.92, TLI = 0.91, RMSEA = 0.07, SRMR = 0.05). The COSMIN guidelines indicate that satisfactory structural validity requires a CFI and TLI greater than 0.95, RMSEA less than 0.06, and SRMR be less than 0.08. Although our CFI, TLI, and RMSEA somewhat failed to satisfy the aforementioned criteria, the results were generally consistent with the COSMIN guidelines. Thus, we believe that the WIPS showed adequate structural validity.

To the best of our knowledge, no scales have focused on and quantified workplace interpersonal problems for care workers working in elder care facilities. Using WIPS to quantitatively assess workplace interpersonal problems among care workers working in elder care facilities, it may be possible to identify those at risk for psychological distress and turnover at an early stage. Workplace interpersonal problems are also related to care workers' job satisfaction and quality of nursing care, and WIPS may be able to assess these indirectly. Future research should examine the clinical utility of using the WIPS to assess workplace interpersonal problems quantitatively and whether the results can be used to improve the work environment, which could prevent care worker turnover and improve care worker mental health and the quality of care received by elderly persons in need of nursing care.

The following limitations of this study should be noted. First, given that Internet users tend to have different sociodemographic and psychological characteristics than do non-users [44], the use of an Internet sample may limit the generalizability of the findings. Second, this study failed to examine criterion validity as recommended by the COSMIN guidelines [24]. As mentioned, workplace interpersonal problems increase the risk of care worker turnover. Should the WIPS be able to capture care worker turnover risk with sufficient sensitivity and specificity, it can certainly be used for care worker turnover prevention measures in elder care facilities. Therefore, criterion validity of the WIPS should be examined in future research on care worker turnover. Finally, the cross-cultural validity recommended by the COSMIN guidelines [24] was not assessed. Second, this study did not consider criterion validity as recommended by the COSMIN guidelines [24]. Given that the WIPS used in the current study was standardized for Japanese care workers, future studies need to utilize the WIPS on non-Japanese care workers and examine its cross-cultural validity in. Although the results of this study confirmed that WIPS has adequate reliability and validity to be used as a scale for assessing workplace interpersonal problems among care workers, it is necessary to examine the points pointed out above to promote the use of WIPS.

## 5 Conclusions

This study developed the WIPS as a scale to assess workplace interpersonal problems among care workers working in elder care facilities using the COSMIN guidelines [23,24] set by the COSMIN group [25]. Notably, our results showed that the WIPS had satisfactory reliability and adequate validity. With the continued growth of the elderly population worldwide, the WIPS is expected to be a useful quantitative measure for assessing workplace interpersonal problems affecting care workers through various aspects.

## Declarations

Approval of the Research Protocol: This study was approved by the Institutional Review Board at the Tottori University Faculty of Medicine (No. 22A070). After explaining the purpose of the study, data were collected only from those who fully understood the contents and had agreed to participate voluntarily. Informed consent was also obtained online from all participants. Registry and the Registration No: N/A. Animal Studies: N/A.

## Funding

This work was supported by 10.13039/501100001691JSPS KAKENHI Grant Number 21K03106.

## CRediT authorship contribution statement

Shinya Takeda: Conceptualization, Methodology, Investigation, Writing – original draft, Writing – review & editing, Visualisation, Project administration, Funding acquisition. Toshiki Fukuzaki: Conceptualization, Methodology, Investigation, Formal analysis, Data curation, Writing – review & editing.

## Declaration of competing interest

The authors declare that they have no known competing financial interests or personal relationships that could have appeared to influence the work reported in this paper.
