# Supplementary material for: Development of the Workplace Interpersonal Problems Scale for Care Workers (WIPS) and examination of its reliability and validity
Source: Heliyon. 2023 Sep 16;9(9):e20156. doi: 10.1016/j.heliyon.2023.e20156 (PMC10559914; doi:10.1016/j.heliyon.2023.e20156)
Supplement: Multimedia component 1 [file mmc1.docx]

Appendix

| While thinking about interpersonal relations at your workplace, please read the following questions and circle the number that most applies to your situation in the past 2 weeks. | Not at all applicable | Not applicable enough | Slightly applicable | Very applicable |
| --- | --- | --- | --- | --- |
| I have not communicated well enough with my coworkers. | 0 | 1 | 2 | 3 |
| I have had difficulties with my coworkers due to differences in our views regarding caregiving and care. | 0 | 1 | 2 | 3 |
| There have been times when I have been ignored by my coworkers. | 0 | 1 | 2 | 3 |
| When a disagreement occurred with my coworkers, I have thought, “This happened because of a person’s bad personality." | 0 | 1 | 2 | 3 |
| I have had difficulties mentoring subordinates or new staff due to disagreements in opinions. | 0 | 1 | 2 | 3 |
| I have felt that I was treated unfairly because my workload differs from that of my coworkers. | 0 | 1 | 2 | 3 |
| I have had difficulties with my coworkers due to disagreements on the future directions at work. | 0 | 1 | 2 | 3 |
| I have overheard my coworkers talking bad about other coworkers, even though I didn't want to hear it. | 0 | 1 | 2 | 3 |
| There have been times when I did not know how to talk to my subordinates or new staff. | 0 | 1 | 2 | 3 |
| I have negatively labeled my coworkers. | 0 | 1 | 2 | 3 |
| I have felt like my opinions are ignored. | 0 | 1 | 2 | 3 |
| I have been frustrated when my workload differed from that of my coworkers due to my supervisor’s inadequate management skills and directions. | 0 | 1 | 2 | 3 |
| I have had difficulties mentoring subordinates or new staff. | 0 | 1 | 2 | 3 |
| I have had difficulties with my coworkers due to differences in perspectives based on rank or post. | 0 | 1 | 2 | 3 |
| I have had a hard time expressing what I wanted to say to my coworkers. | 0 | 1 | 2 | 3 |
| I have been unreasonably blamed by my coworkers in a strong tone of voice. | 0 | 1 | 2 | 3 |
| I have had difficulties with my coworkers due to differences in perspectives because of an age gap. | 0 | 1 | 2 | 3 |
| When a disagreement occurred with my coworkers, I have thought, "This happened because I am not fond of this person." | 0 | 1 | 2 | 3 |
| I have felt that I was the only person working hard while some coworkers were just relaxing. | 0 | 1 | 2 | 3 |
| I have had difficulties due to differences in values between myself and my subordinates or new staff. | 0 | 1 | 2 | 3 |
| There are times when I did not feel comfortable asking questions or consulting with my supervisors or coworkers. | 0 | 1 | 2 | 3 |
| When a disagreement occurred with my coworkers, I have thought, “This happened because I didn’t get along with this person.” | 0 | 1 | 2 | 3 |
| There have been times when my coworkers talked to me in a passive-aggressive manner. | 0 | 1 | 2 | 3 |
